# Low-keV Virtual Monoenergetic Imaging for Bronchial Artery Visualization on Photon-Counting Detector Computed Tomography

**DOI:** 10.3390/diagnostics15111354

**Published:** 2025-05-28

**Authors:** Xuyang Sun, Tetsu Niwa, Takakiyo Nomura, Ryoichi Yoshida, Kazuo Koyanagi, Jun Hashimoto

**Affiliations:** 1 Department of Diagnostic Radiology, Tokai University School of Medicine, 143 Shimokasuya, Isehara 259-1193, Japan; xxxuyangsun@gmail.com (X.S.); yh_4amm1208@yahoo.co.jp (T.N.); junhashi@tokai.ac.jp (J.H.); 2 Department of Radiology, Tokai University Hospital, 143 Shimokasuya, Isehara 259-1193, Japan; r.yoshida@tokai.ac.jp; 3 Department of Gastroenterological Surgery, Tokai University School of Medicine, 143 Shimokasuya, Isehara 259-1193, Japan; kkoyanagi@tokai.ac.jp

**Keywords:** computed tomography, photon-counting detector computed tomography, bronchial artery visualization, virtual monoenergetic imaging, contrast-enhanced computed tomography

## Abstract

**Background/Objectives:** This study aims to determine the optimal use of virtual monoenergetic imaging (VMI) for visualizing the bronchial artery on photon-counting detector computed tomography (PCD-CT). **Methods:** We evaluated the visibility of the bronchial artery on PCD-CT in 34 consecutive patients with esophageal cancer (twenty-eight men, six women; mean age, 70.2 years) prior to surgery. Region-of-interest measurements were taken at the right bronchial artery at the tracheal bifurcation level, mediastinal fat, and the erector spinae muscles on contrast-enhanced early-phase CT. We compared the CT attenuation of the bronchial artery, image noise, and contrast-to-noise ratio (CNR) across VMI at 40, 50, 60, and 70 keV. Additionally, two radiologists performed a subjective image quality assessment by comparing VMI at 40, 50, and 60 keV with 70 keV, rating bronchial artery enhancement, border clarity, peripheral visibility, and image noise. **Results:** CT attenuation, image noise, and CNR significantly differed across VMI energy levels (*p* < 0.00001). Lower-keV VMI demonstrated higher CT attenuation and increased noise but also higher CNR (all *p* < 0.05). Both radiologists rated bronchial artery enhancement, border clarity, and peripheral visibility higher at 40 and 50 keV than at 70 keV, with the highest scores observed at 40 keV (all *p* < 0.05). Observer 1 noted slightly increased noise at 40 and 50 keV, while observer 2 observed this effect at 40 keV compared with 70 keV. **Conclusions:** Low-keV (40–50 keV) VMI on PCD-CT enhances bronchial artery visualization.

## 1. Introduction

The bronchial arteries typically originate from the thoracic aorta, traverse the mediastinum, and branch into the trachea, bronchi, hilar lymph nodes, mid-esophagus, and other structures, where they form capillaries to supply nutrients [1]. However, the bronchial arteries exhibit considerable anatomical variation in terms of number, origin, distribution, and course among individuals [2,3,4]. Understanding their anatomical trajectory is essential, particularly in thoracic surgery, bronchial artery embolization for massive hemoptysis, and interventional radiologic procedures for lung tumors [2,5,6,7]. With the increasing prevalence of thoracic surgeries, various surgical interventions on thoracic organs may affect the bronchial arteries in distinct ways [8].

In routine clinical practice, the bronchial artery is typically evaluated noninvasively using contrast-enhanced computed tomography (CT). Due to the small size of the bronchial artery, strong contrast enhancement is necessary for its visualization on CT. One approach for achieving this is the use of low-keV images in virtual monoenergetic imaging (VMI). Since the introduction of dual-energy CT, VMI has been widely utilized, as it applies X-ray imaging at two distinct tube voltages [9,10]. VMI is generated through an image-based monoenergetic algorithm applied to data from dual-energy CT scans, allowing image reconstruction at arbitrary keV levels. A key feature of VMI is that low-keV images increase the CT attenuation of contrast materials, potentially improving vessel delineation [9,11]. However, lower-keV VMI also tends to increase image noise [10]. Therefore, optimizing the balance between vascular contrast and image noise is crucial, with VMI at 60–70 keV generally considered appropriate for vascular imaging in dual-energy CT [8,12].

The recently introduced clinically available photon-counting detector (PCD)-CT directly converts X-ray photons into electrical signals [13,14,15,16,17,18,19,20]. Unlike conventional CT, which uses an energy-integrating detector (EID) system, PCD-CT lacks a reflective layer between detectors, allowing for high spatial resolution imaging [21,22]. PCD-CT can count individual incident photons and determine their energy levels, enabling simultaneous multi-energy imaging, including VMI, virtual non-contrast imaging, and iodine mapping [13,23,24,25,26,27]. Additionally, PCD-CT reduces electronic noise by discriminating between scattered radiation and primary photons and by setting the low-energy threshold above the electronic noise amplitude, thereby enhancing the contrast-to-noise ratio (CNR) [14,16,17] and lowering the radiation dose [13,28]. These techniques have been used in clinical applications, including ultra-high-resolution bone imaging, enhanced contrast agent visibility for blood vessels and tumors, improved detectability of hemorrhage in contrast-enhanced CT, analysis of blood flow, and detection of gout [10,14,19]. Recent studies have demonstrated the effectiveness of low-keV VMI in vascular imaging, including applications for the aorta [29] and abdominal arteries [30,31].

We hypothesized that PCD-CT could optimize the use of low-keV VMI, enhancing the visualization of relatively small arteries such as the bronchial artery. A better understanding of the bronchial artery’s anatomy before thoracic surgery and interventional radiologic procedures is crucial. This study aimed to determine the optimal VMI settings on PCD-CT for visualizing the bronchial arteries.

## 2. Materials and Methods

### 2.1. Patients

PCD-CT was performed as the initial CT examination for esophageal cancer prior to surgery. This study included 34 consecutive patients (28 men, 6 women; mean age, 70.2 years; age range, 43–87 years) who underwent scanning between July 2022 and January 2024. Their CT images were retrospectively analyzed. The study was approved by the Institutional Review Board for Clinical Research at Tokai University, with a waiver of informed consent (IRB No. 23R211).

### 2.2. CT Image Acquisition

CT imaging was performed using a dual-source PCD-CT (NAEOTOM Alpha; Siemens Healthineers, Forchheim, Germany) with the following scan parameters: tube voltage, 120 kV; collimation, 144 × 0.4 mm; pitch, 2.4; gantry rotation time, 0.5 s; and CARE keV image quality level, 80.

Dual-phase contrast-enhanced CT was performed using a contrast medium dose based on the patient’s body weight (<57 kg, 300 mg I/mL; 57–66 kg, 320 mg I/mL; and >66 kg, 370 mg I/mL). The contrast agent was administered via a 20-gauge needle inserted into the antecubital vein using an injector (DUAL SHOT GX7; Nemoto Kyorindo, Tokyo, Japan) at a rate of 23.0 mg I/body weight (kg)/s for 20 s. Early-phase scan images were acquired using a bolus-tracking method to determine the optimal timing by placing a region of interest (ROI) in the descending aorta at the level of the tracheal bifurcation. Scanning started 15 s after the administration of the contrast agent. The early-phase scan began 5 s after the CT attenuation in the ROI reached 180 HU, while late-phase images were obtained 2 min after contrast injection. The early-phase scan covered the area from the top of the chest to the diaphragm, whereas the late-phase scan extended from the neck to the pelvis. Early-phase CT images were reconstructed with a thickness of 0.4 mm and an interval of 0.4 mm, using the Qr40 kernel and quantum iterative reconstruction (QIR) at strength level 2 and a 512 × 512 matrix size (Table 1). The images were transferred to a workstation (syngo.via, 8.04, Siemens Healthcare, Forchheim, Germany) for analysis.

### 2.3. Objective Image Analysis

CT images were displayed on the workstation with a slice thickness of 1 mm. CT attenuation and standard deviation (SD) were measured at the following locations by placing ROIs: the right bronchial artery, mediastinal fat, and erector spinae muscle at the level of the tracheal bifurcation. Initially, ROIs were placed on VMI at 40 keV, and then the keV value was sequentially adjusted to 50, 60, and 70 keV while maintaining the same ROI to measure CT attenuation and SD. All measurements were performed by a board-certified radiologist (T.N. (Tetsu Niwa)) with 26 years of experience in chest radiology. The CNR was calculated using the following formula:

CNR = (CT attenuation of the bronchial artery − CT attenuation of the muscle)/noise

where noise was defined as the SD of the CT attenuation of the mediastinal fat. Measurements were conducted twice, with a 1-month interval, and the final values were obtained by averaging the two sets of measurements.

### 2.4. Subjective Image Analysis

Subjective image analysis was performed by two board-certified radiologists (T.N. (Tetsu Niwa) and T.N. (Takakiyo Nomura), with 11 years of experience in chest radiology). The image quality of VMIs at 40, 50, and 60 keV was compared with that at 70 keV. VMI at 70 keV was used as the reference for contrast-enhanced CT, as multiple vendors consider it to provide attenuation values equivalent to conventional images [32,33]. On the workstation, a randomly selected image from VMI at 40, 50, and 60 keV was displayed on the left side of the monitor, with patient information and keV parameters hidden, while the corresponding 70 keV VMI for the same patient was displayed on the right as a reference. All images were initially set to a window width of 400 HU and a window level of 60 HU. Observers could adjust these settings independently for each image. CT images were displayed with a 1 mm slice thickness, and synchronized slices from low-keV and 70-keV VMIs were used to allow simultaneous comparison. Observers independently rated the image quality based on arterial enhancement, border clarity, peripheral visualization of the right bronchial artery, and image noise while remaining blinded to patient information and imaging parameters. They were only aware that the right-side images served as references. The evaluation criteria were as follows: bronchial artery enhancement (1, prominently less; 2, slightly less; 3, same level as the reference; 4, slightly better; and 5, prominently better), bronchial artery border clarity (1, obviously worse; 2, slightly worse; 3, same level as the reference; 4, slightly more distinct; and 5, obviously more distinct), peripheral visibility of the bronchial artery (1, prominently less; 2, slightly less; 3, same level as the reference; 4, slightly better; and 5, prominently better), and image noise (1, obviously more; 2, slightly more; 3, same level as the reference; 4, slightly less; and 5, prominently less).

### 2.5. Representative Image

Representative partial maximum intensity projection (MIP) images were generated to display the bronchial artery at different keV settings of VMI.

### 2.6. Statistical Analysis

The Kolmogorov–Smirnov test showed that the data did not follow a normal distribution (all *p* > 0.05). CT attenuation, noise, and CNR for VMI at 40, 50, 60, and 70 keV were compared using the Friedman test, followed by post hoc pairwise comparisons (Conover’s method). The increase rates of CT attenuation, noise, and CNR for VMI at 40, 50, and 60 keV (Value40–60 keV) relative to those at 70 keV (Value70 keV) were calculated using the following formula:

Increase rate = (Value40–60 keV − Value70 keV)/Value70 keV × 100 (%)

The subjective scores for bronchial artery enhancement, border clarity, peripheral visibility, and image noise at 40, 50, and 60 keV were compared using the Friedman test, followed by post hoc pairwise comparisons (Conover’s method).

Inter-rater agreement was assessed using Gwet’s AC1 due to score frequency imbalances. Gwet’s AC1 was calculated using R statistical software (version 4.3.1; R Foundation, Vienna, Austria) and the irrCAC package, which was regarded as follows: slight (0.01–0.20), fair (0.21–0.40), moderate (0.41–0.60), good (0.61–0.80), and almost perfect (0.81–1.0).

Statistical analysis was conducted using MedCalc Statistical Software version 22.030 (MedCalc Software Ltd., Ostend, Belgium; https://www.medcalc.org), with a *p* value of <0.05 considered statistically significant.

## 3. Results

The mean volume CT dose index volume (CTDIvol) was 4.34 mGy (range, 3.09–6.48).

### 3.1. Objective Image Analysis

**Figure 1:**
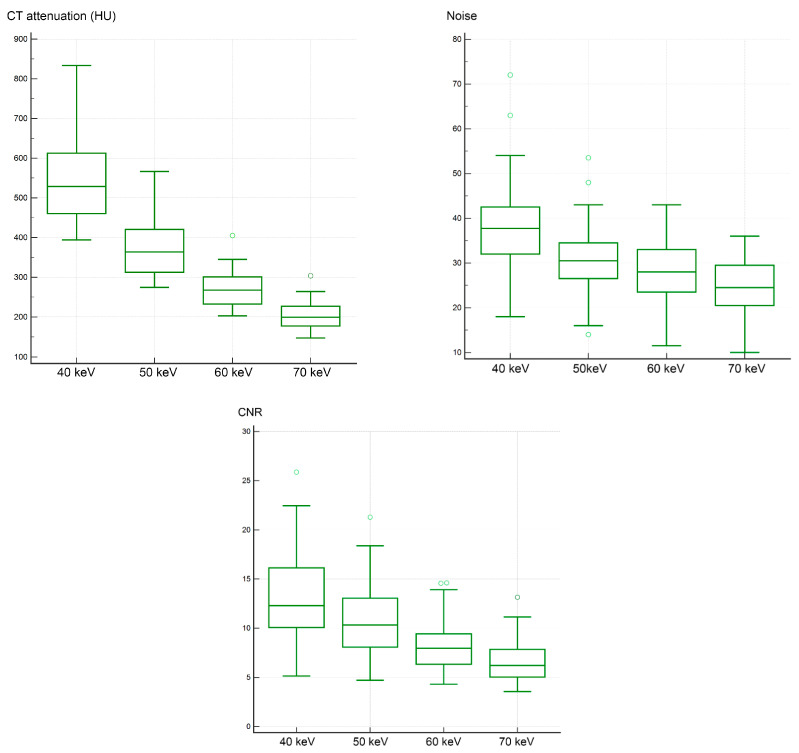
Comparison of CT attenuation, noise, and contrast-to-noise ratio among VMI at 40–70 keV. Data are presented as box-and-whisker plots, indicating the median, upper and lower quartiles, and the maximum and minimum values, excluding outliers. Circles represent outliers, defined as values smaller than the lower quartile minus 1.5 times the interquartile range or larger than the upper quartile plus 1.5 times the interquartile range.

The median CT attenuation (range) of the bronchial artery for VMI at 40, 50, 60, and 70 keV was 528.8 (394.0–833.5), 363.8 (274.5–566.5), 267.5 (203.0–405.0), and 199.5 (147.5–303.5) HU, respectively. These values showed significant differences (*p* < 0.00001). Post hoc tests revealed significant differences in CT attenuation between each keV VMI (*p* < 0.05). Lower-keV VMI resulted in higher CT attenuation (Figure 1). The mean ± SD of the increase rates in CT attenuation for VMI at 40, 50, and 60 keV compared with 70 keV were 170.4% ± 38.5%, 85.0% ± 19.8%, and 33.5% ± 8.2%, respectively. The median noise (range) for VMI at 40, 50, 60, and 70 keV was 37.8 (18.0–72.0), 30.5 (14.0–53.5), 28.0 (11.5–43.0), and 24.5 (10.0–36.0), respectively, showing significant differences (*p* < 0.0001). Post hoc tests revealed significant differences in noise between each keV VMI (*p* < 0.05). Lower-keV VMI resulted in higher noise (Figure 1). The mean ± SD of the increase rates in noise for VMI at 40, 50, and 60 keV compared with 70 keV were 55.6% ± 27.4%, 26.5% ± 18.7%, and 11.6% ± 10.4%, respectively. The median CNR (range) of the bronchial artery for VMI at 40, 50, 60, and 70 keV was 12.3 (5.1–25.9), 10.3 (4.7–21.3), 8.0 (4.3–14.6), and 6.2 (3.6–13.1), respectively, with significant differences observed (*p* < 0.0001). Post hoc tests revealed significant differences in CNR between each keV VMI (*p* < 0.05). Lower-keV VMI resulted in higher CNR values (Figure 1). The mean ± SD of the increase rates in CNR for VMI at 40, 50, and 60 keV compared with 70 keV were 109.6% ± 53.5%, 68.0% ± 35.7%, and 28.7% ± 15.5%, respectively. The results are summarized in Table 2.

### 3.2. Subjective Image Analysis

**Figure 2:**
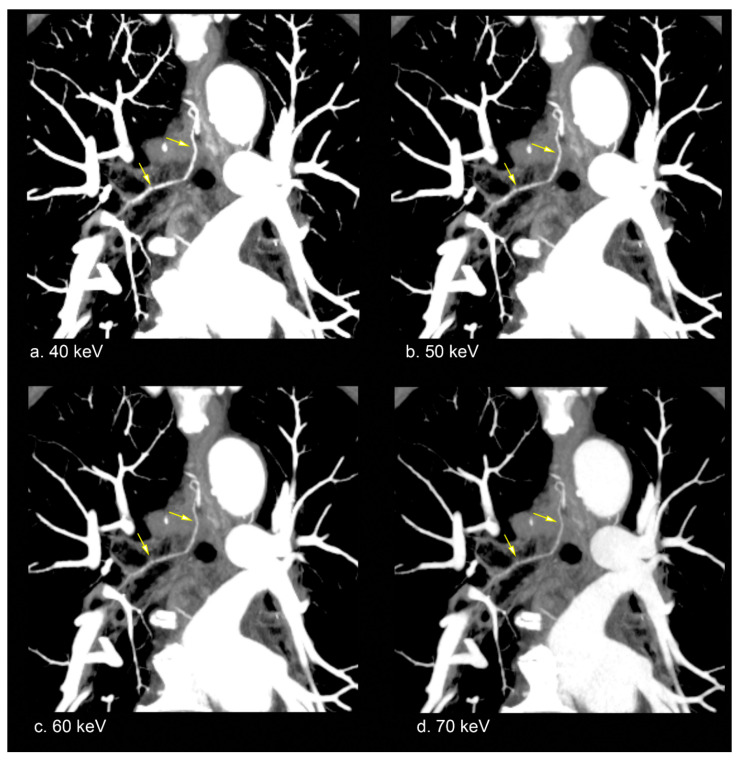
Representative VMI images of the bronchial artery (arrows) at 40 keV (**a**), 50 keV (**b**), 60 keV (**c**), and 70 keV (**d**) in an 84-year-old man with esophageal cancer, displayed using partial MIP. Greater contrast enhancement of the bronchial artery is observed in lower-keV VMI.

**Figure 3:**
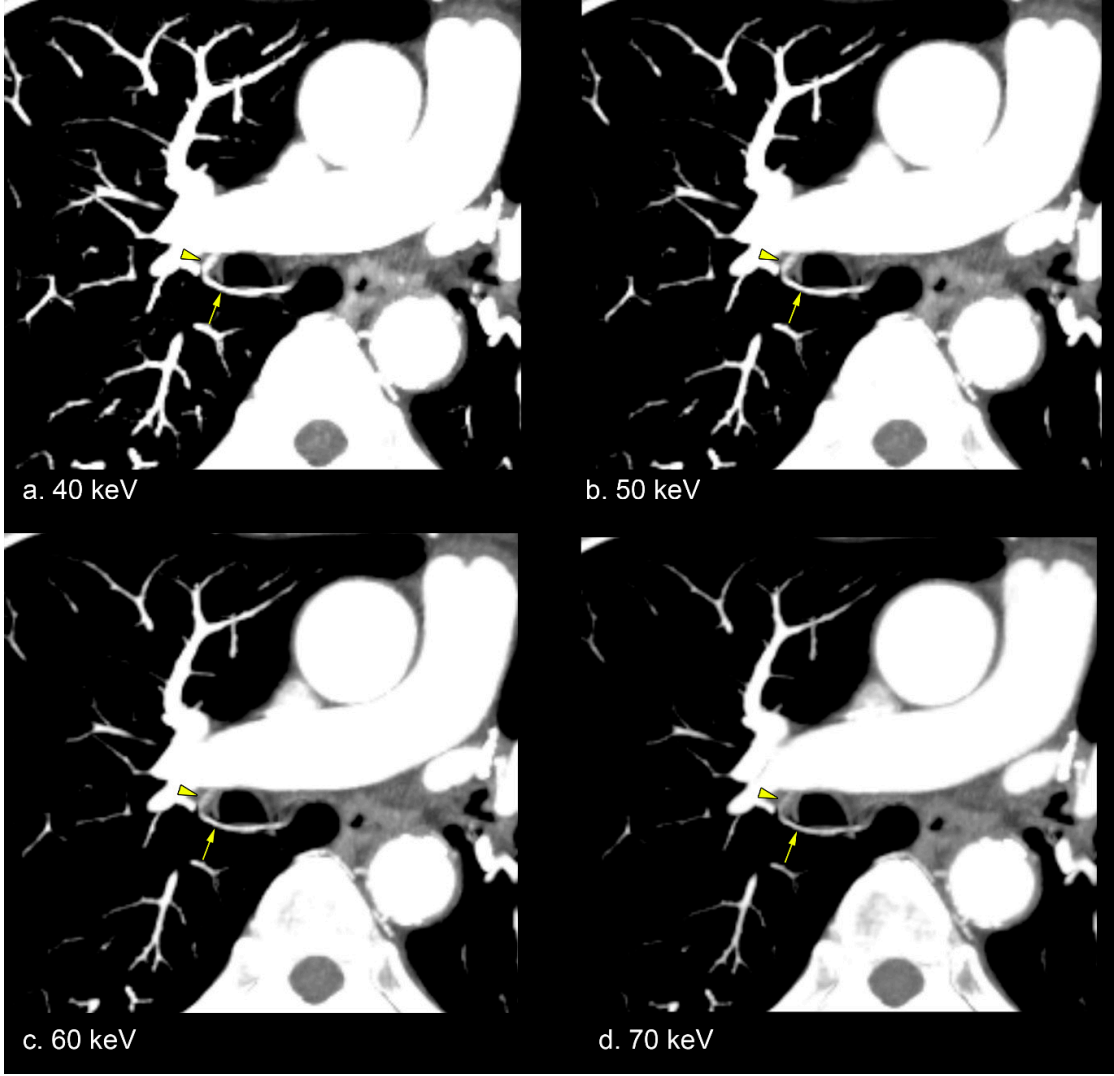
Representative VMI images of the bronchial artery (arrows) at 40 keV (**a**), 50 keV (**b**), 60 keV (**c**), and 70 keV (**d**) in a 71-year-old man with esophageal cancer, displayed using partial MIP. Enhanced contrast of the bronchial artery (arrows) is evident in lower-keV VMI. The peripheral portion of the bronchial artery (arrowheads) is also better visualized at lower-keV VMI.

Table 3 presents subjective assessment scores from two observers. Both observers rated bronchial artery enhancement higher for VMI at 40, 50, and 60 keV compared with VMI at 70 keV (median scores: 5, 5, and 4 for observer 1; 5, 4, and 4 for observer 2). The differences in enhancement scores across these keV levels were statistically significant for both observers (*p* < 0.00001). Post hoc analysis indicated that VMI at 40 keV had the highest scores, followed by 50 and 60 keV (all *p* < 0.05; Figure 2). For bronchial artery border clarity, observer 1 assigned higher scores to VMI at 40, 50, and 60 keV (median scores: 5, 5, and 4, respectively), while observer 2 gave higher ratings to VMI at 40 and 50 keV (median scores: 5 and 4, respectively) compared with VMI at 70 keV. These differences were statistically significant for both observers (*p* < 0.00001), with post hoc analysis showing that VMI at 40 keV received the highest scores, followed by 50 and 60 keV (all *p* < 0.05). Peripheral visibility of the bronchial artery was also rated higher for VMI at 40 and 50 keV (median scores: 5 and 4, respectively) by both observers compared with VMI at 70 keV. The differences in peripheral visibility scores among VMI at 40, 50, and 60 keV were statistically significant for both observers (*p* < 0.00001). Post hoc analysis showed that VMI at 40 keV had the highest scores, followed by 50 and 60 keV (all *p* < 0.05; Figure 3). Slightly increased noise was noted at VMI 40 and 50 keV (median scores: 2 and 2, respectively) by observer 1 and at VMI 40 keV (median score: 2) by observer 2. Noise scores for VMI at 40, 50, and 60 keV were significantly different for both observers (*p* < 0.00001). Post hoc analysis revealed that observer 1 assigned the lowest scores to VMI at 40 keV, followed by 50 and 60 keV (all *p* < 0.05). Similarly, observer 2 rated the noise score on VMI at 40 keV lower than at 50 and 60 keV (*p* < 0.05).

Inter-rater agreement for each VMI was moderate to almost perfect for bronchial artery enhancement and border clarity (AC1: 0.52–0.82 and 0.52–0.85, respectively), fair to good for peripheral visibility (AC1: 0.35–0.76), and fair to moderate for noise (AC1: 0.26–0.38).

## 4. Discussion

Low-keV VMI demonstrated superior image quality for the visualization of the bronchial artery.

### 4.1. Objective Image Analysis

Our findings indicate that CT attenuation values were higher at lower-keV VMI, which aligns with the established principle that CT attenuation value increases as keV decreases in VMI [15]. High CT attenuation in contrast-enhanced CT is crucial for identifying and assessing the course of the bronchial artery, which is often small. From this perspective, low-keV VMI, such as 40 keV, appears beneficial. However, lower-keV VMI is also associated with increased image noise. In this study, greater noise was observed at lower-keV VMI, consistent with previous research [34]. However, while noise increased at lower keV, the increase was relatively controlled in PCD-CT, whereas the CT attenuation value showed a pronounced rise. Notably, the increase rate in CT attenuation at 40 keV VMI was significantly greater than the corresponding increase rate in noise at the same keV level. This contributed to a high CNR at lower keV despite the noise increase. In dual-energy EID-CT, lower-keV VMI (e.g., 40–50 keV) has been considered unsuitable for contrast-enhanced imaging of relatively small arteries [8,35] due to substantial image noise. Conversely, our findings suggest that even lower-keV VMI can be useful for visualizing small arteries in PCD-CT, likely due to the improved noise reduction capability of PCD, which directly converts X-ray photons into electrical signals. Additionally, previous studies have reported a decrease in CT attenuation of fat at lower-keV VMI [36], which may further enhance the contrast between the bronchial artery and the surrounding mediastinal fat, improving visualization in low-keV VMI.

### 4.2. Subjective Image Analysis

In our subjective analysis, both observers tended to assign higher scores for bronchial artery enhancement, border clarity, and peripheral visibility on VMI at 40 and 50 keV. These scores increased as keV decreased. The improved visibility of the bronchial artery at lower-keV VMI is likely due to the higher CT attenuation observed in the objective analysis. As bronchial arteries are typically small, their recognition relies on strong contrast enhancement, particularly for peripheral branches. Although noise was slightly higher at lower-keV VMI, the differences in noise scores between 40–60 keV VMI and 70 keV VMI were relatively minor. This indicates that the increase in noise at lower keV remained controlled, which is consistent with the objective analysis results. Despite the noise increase, visibility scores for the bronchial artery remained high, suggesting that the noise level at low-keV VMI did not significantly hinder bronchial artery recognition. Bronchial arteries travel through mediastinal fat and alongside bronchi, both of which typically have low CT attenuation. Therefore, noise in these structures likely had minimal impact on bronchial artery visibility. In this study, 70 keV VMI was used as the reference for contrast-enhanced CT, as it is considered to provide attenuation values comparable to conventional imaging [32,33]. Higher-keV VMIs (particularly those above 80 keV) were not evaluated owing to their known lower contrast enhancement [30], making them less useful for bronchial artery assessment.

Inter-rater agreements varied, ranging from fair to almost perfect. Although the inter-rater agreement was relatively good for bronchial artery enhancement and border clarity, the agreement for noise was considered fair to moderate. This suggests that image impression varies among individuals, particularly regarding image noise. However, both readers demonstrated a consistent tendency to correlate image scores with VMI energy levels. Therefore, we believe that this study provides a reliable perspective on the use of VMI for PCD-CT.

### 4.3. Utility of Low-keV VMI for Bronchial Artery

Our subjective and objective analyses demonstrated that using low-keV VMI improved bronchial artery visibility by enhancing contrast while maintaining a relatively controlled noise level. This finding aligns with previous studies on the utility of VMI in PCD-CT, where low-keV VMI was reported to be beneficial for imaging the aorta at 45–50 keV [29] and abdominal arteries at 50–70 keV [30,31]. For bronchial artery visualization, we suggest that 40–50 keV VMI is optimal, particularly for thinner segments, as these low-keV VMIs demonstrated higher CNR and subjective assessment scores. Ma et al. [8] evaluated bronchial artery visibility using VMI in dual-energy EID-CT and found that an optimal setting was approximately 63 keV. However, in PCD-CT, even lower-keV VMI can be effectively used, likely due to the superior noise reduction capability of PCD compared with dual-energy EID-CT. Dillinger et al. [30] assessed VMI for abdominal vessels and concluded that 60–70 keV VMI provided the best image quality. They reported lower CNR at 40–50 keV VMI compared with 60–70 keV and noted that low-keV VMI caused the blooming of the contrast agent, which obscured vessel wall delineation. In contrast, our study found higher CNR at 40–50 keV VMI for the bronchial artery. This discrepancy may stem from differences in CNR calculation methods. Despite some degree of blooming, strong contrast enhancement improves bronchial artery identification and assessment, which is essential in clinical practice due to the small size of these arteries. VMI also allows real-time keV adjustment on the workstation, enabling users to modify the setting if 40 keV VMI presents excessive noise, achieving an optimal balance between vessel enhancement and noise levels.

Our study found that low-keV VMI in PCD-CT provides clear enhancement of bronchial arteries, making it a valuable tool for preoperative assessment of their number and course. This information is crucial for planning and executing treatment. Understanding the bronchial artery anatomy before esophageal cancer surgery is particularly important, as preoperative evaluation helps prevent intraoperative arterial injury and reduces the risk of complications. Additionally, low-keV VMI may aid in planning interventional radiologic treatments involving the bronchial arteries, such as those for hemoptysis or mediastinal hematoma [37,38]. In this study, we used high-pitch image acquisition to reduce motion artifacts from the heart. Although high-pitch scanning may reduce image quality, dual-source scanning has the potential to enhance overall image quality.

### 4.4. Comparison to EID-CT

Previous studies have shown that PCD-CT outperforms EID-CT in various applications, including vascular, cardiac, abdominal, thoracic, central nervous system, and brain imaging [13,39,40,41,42,43]. However, we did not compare PCD-CT with EID-CT in this study. Notably, PCD-CT demonstrates greater potential than conventional CT, particularly at low keV [44]. If its ability to improve image quality is further validated, PCD-CT could help reduce radiation doses [13]. In fact, CTDIvol in the present study (mean, 4.34 mGy) was lower than in a previous study regarding EID-CT analysis for bronchial artery (7.7 mGy) [8]. Furthermore, the high-resolution detector of PCD-CT is advantageous for visualizing fine blood vessels, suggesting continued expansion in clinical use.

### 4.5. Limitations

This study has several limitations. First, the sample size was relatively small. Additionally, all CT images were obtained from patients with esophageal cancer, which may introduce selection bias. Second, the analysis was limited to VMIs at 40–70 keV, as higher-keV VMIs were considered to have low contrast enhancement, making them less suitable for evaluating the bronchial artery. Furthermore, reconstruction parameters such as kernel and QIR strength level were not examined. Noise evaluation might have been influenced by window width and level settings. Third, no external reference was used to assess bronchial artery visibility; instead, visibility was compared only among VMIs at different keV levels.

## 5. Conclusions

Low-keV VMI in PCD-CT is useful for evaluating the course and number of bronchial arteries in preoperative patients. Although increased noise is observed, the substantial increase in CT attenuation at low keV (40–50 keV) VMI contributes to improved visibility of the bronchial artery. PCD-CT enhances the diagnostic value of preoperative bronchial artery assessment.

## Figures and Tables

**Table 1:** CT scanning and reconstruction parameters.

Scan Parameter	Value
Tube voltage	120 kv
Collimation	144 × 0.4 mm
Pitch	2.4
Gantry rotation time	0.5 s
CARE keV image quality level	80
Reconstruction kernel	Qr40
QIR	Strength level 2
Matrix size	512 × 512

**Table 2:** Objective analysis of bronchial artery. Data are presented as median (range).

	VMI at 40 keV	VMI at 50 keV	VMI at 60 keV	VMI at 70 keV	*p*
CT attenuation (HU)	528.8 (394.0–833.5)	363.8 (274.5–566.5)	267.5 (203.0–405.0)	199.5 (147.5–303.5)	<0.00001 *
Noise	37.8 (18.0–72.0)	30.5 (14.0–53.5)	28.0 (11.5–43.0)	24.5 (10.0–36.0)	<0.00001 *
CNR	12.3 (5.1–25.9)	10.3 (4.7–21.3)	8.0 (4.3–14.6)	6.2 (3.6–13.1)	<0.00001 *

* Post hoc tests revealed significant differences between each keV VMI (*p* < 0.05).

**Table 3:** Subjective image assessment scores for bronchial artery enhancement, border clarity, peripheral visibility, and image noise for VMI at 40–60 keV compared with VMI at 70 keV. Data represent the number of patients in each score.

Assessment	VMI at 40 keV					VMI at 50 keV					VMI at 60 keV					*p*
Score	1	2	3	4	5	1	2	3	4	5	1	2	3	4	5	
Enhancement																
Observer 1	0	0	0	3	31	0	0	0	10	24	0	0	11	19	4	<0.00001 *
Observer 2	0	0	0	7	27	0	0	1	23	10	0	0	14	19	1	<0.00001 *
Border clarity																
Observer 1	0	0	0	2	32	0	0	0	10	24	0	0	8	23	3	<0.00001 *
Observer 2	0	0	0	7	27	0	0	3	22	9	0	0	19	14	1	<0.00001 *
Peripheral visibility																
Observer 1	0	0	0	5	29	0	0	3	15	16	0	0	20	11	3	<0.00001 *
Observer 2	0	0	0	6	28	0	0	3	21	10	0	0	24	9	1	<0.00001 *
Noise																
Observer 1	9	24	1	0	0	3	22	9	0	0	1	10	23	0	0	<0.00001 *
Observer 2	3	16	15	0	0	0	4	30	0	0	0	3	31	0	0	<0.00001 ^†^

* Post hoc tests revealed significant differences between each keV VMI (*p* < 0.05). ^†^ Post hoc tests revealed significantly lower scores on 40 keV than those on 50 and 60 keV.

## Data Availability

The data supporting this study’s findings are available from the corresponding author upon reasonable request. The data are not publicly available due to privacy.

## Abbreviations

The following abbreviations are used in this manuscript:

CNR	Contrast-to-noise ratio
CT	Computed tomography
EID	Energy-integrating detector
MIP	Maximum intensity projection
PCD	Photon-counting detector
ROI	Regions of interest
SD	Standard deviation
VMI	Virtual monoenergetic imaging
QIR	Quantum iterative reconstruction
